# Effects of Different Dialysis Strategies on Inflammatory Cytokine Profile in Maintenance Hemodialysis Patients with COVID-19: A Randomized Trial

**DOI:** 10.3390/jcm10071383

**Published:** 2021-03-30

**Authors:** Pasquale Esposito, Leda Cipriani, Daniela Verzola, Maria Antonietta Grignano, Mara De Amici, Giorgia Testa, Fabrizio Grosjean, Elisa Russo, Giacomo Garibotto, Teresa Rampino, Francesca Viazzi

**Affiliations:** 1 Department of Internal Medicine, University of Genova, 16132 Genova, Italy; cipriani.leda@gmail.com (L.C.); daverz@libero.it (D.V.); elisa24russo@gmail.com (E.R.); gari@unige.it (G.G.); francesca.viazzi@unige.it (F.V.); 2 IRCCS Ospedale Policlinico San Martino, Clinica Nefrologica, Dialisi, Trapianto, 16132 Genova, Italy; 3 Unit of Nephrology, Dialysis and Transplantation, Fondazione IRCCS Policlinico San Matteo, and University of Pavia, 27100 Pavia, Italy; wta87@hotmail.it (M.A.G.); f.grosjean@smatteo.pv.it (F.G.); teresa.rampino@smatteo.pv.it (T.R.); 4 Laboratory of Immuno-Allergology of Clinical Chemistry and Pediatric Clinic, Fondazione IRCCS Policlinico San Matteo, 27100 Pavia, Italy; m.deamici@smatteo.pv.it; 5 Pediatrics Clinic, Fondazione IRCCS Policlinico San Matteo, University of Pavia, 27100 Pavia, Italy; g.testa@smatteo.pv.it

**Keywords:** COVID-19, hemodialysis, expanded hemodialysis, protein-leaking dialyzer, cytokines, inflammaging

## Abstract

Uncontrolled inflammation plays a relevant role in the pathogenesis of coronavirus disease-19 (COVID-19). Here, we studied the time trend of inflammatory markers in a population of hemodialysis (HD) patients affected by COVID-19, undergoing two different dialysis approaches. In a prospective study, thirty-one maintenance HD patients with COVID-19 were randomized to expanded HD (HDx), performed using a medium cut-off membrane, or standard treatment using a protein-leaking dialyzer (PLD). Circulating levels of interleukin-6 (IL-6), interleukin-8 (IL-8), interleukin-10 (IL-10), soluble TLR4 (sTLR4), and interferon-gamma (IFN-γ), were collected at diagnosis, and one and two weeks after. Compared with 14 non-infected HD patients, COVID-19 patients showed lymphopenia and higher ferritin and lactate dehydrogenase levels. Moreover, COVID-19 patients had higher levels of IL-10 (15.2 (12.5) vs. 1.2 (1.4) pg/mL, *p* = 0.02). Twenty-nine patients were randomized to HDx (n = 15) or PLD (n = 14). After a single treatment, IL-8 showed a significant reduction in both groups, whereas IL-10 decreased only in HDx. All over the study, there were no significant modifications in circulating cytokine levels between the two groups, except for a parallel increase of IL-8 and IL-10 at one week control in the HDx group. No correlations were found between cytokine levels and clinical outcomes. In maintenance HD patients, COVID-19 is not related to a sustained inflammatory response. Therefore, modulation of inflammation seems not to be a suitable therapeutic target in this specific population.

## 1. Introduction

The current coronavirus disease-19 (COVID-19) pandemic is caused by infection by severe acute respiratory syndrome coronavirus 2 (SARS-CoV-2) [1]. It presents a variable clinical picture from asymptomatic infection to severe pneumonia and multi-organ failure [2]. The study of the pathogenesis of COVID-19 is a rapidly evolving field, and evidence is increasing about the complexity of the disease and the involvement of different pathogenic mechanisms. In particular, while direct viral invasion seems to initiate the pathological process, secondary mechanisms may play a crucial role in expanding systemic and local injury [3]. This is a case of uncontrolled inflammation, which is related to disease severity and unfavorable outcomes [4]. These findings provided a rational basis for using anti-inflammatory drugs and extracorporeal procedures with a high capacity of cytokine adsorption and removal in the treatment of COVID-19 [5,6]. Clinically, COVID-19 may involve patients of all ages, but it appears particularly severe in old subjects with pre-existent health problems. In this regard, patients with chronic kidney disease (CKD), and in particular those on hemodialysis (HD), are at high risk for COVID-19 complications because most of them are old, frail, and have multiple comorbidities [7]. Epidemiological and clinical studies confirmed this increased risk, so that impairment of renal function is considered an independent risk factor for unfavorable outcomes [8,9]. Pathogenesis of COVID-19 related morbidity in HD patients is unclear; in particular, little data have been reported on the inflammatory response of HD patients affected by COVID-19. In an interesting paper by Ma et al., the authors found that HD patients affected by COVID-19 had lower values of lymphocytes and inflammatory cytokines compared with non-HD infected patients [10]. This is a crucial point since it is well-known that uremic patients present with multifaceted immune dysfunction, involving both innate and adaptive immune responses, in which the clinical counterpart is the high susceptibility to infection and malignancy, and resistance to vaccination [11]. Therefore, it is conceivable that an impaired immune system can impact the type and extent of the inflammatory response during COVID-19. On the other hand, as for the general population, in HD patients, accentuated inflammation elicited by COVID-19 may be a determinant of clinical outcomes. Therefore, modulation of the inflammatory response could potentially constitute a therapeutic target. Interestingly, previous experiences have shown the possibility of using dialysis as an immunomodulator therapy [12,13]. Different dialysis approaches may be used for this purpose in maintenance HD patients. Expanded HD (HDx) is a dialysis modality based on medium cut-off (MCO) membranes, which, in comparison with standard dialysis membranes, provide better removal of solutes of molecular weight up to 45 kDa, including inflammatory cytokines and uremic toxins [14,15]. A different strategy, based on the use of the so-called protein-leaking dialyzers (PLD), taking advantage of an accentuated membrane absorption capacity, can remove high-molecular-weight molecules, such as inflammatory solutes [16]. With this study, we aimed to describe and characterize circulating levels of inflammatory cytokines and their time trend by evaluating the effects of two different dialysis approaches (i.e., HDx vs. PLD) in a population of maintenance HD patients affected by COVID-19.

## 2. Materials and Methods

### 2.1. Study Design and Patients

For this prospective randomized study, we recruited maintenance hemodialysis patients with confirmed COVID-19 infection from March 16th to April 20th, 2020, enrolled in San Martino, University Hospital of Genoa, Italy, and San Matteo, University Hospital of Pavia, Italy. Nasopharyngeal swabs for SARS-CoV-2 were performed on each HD patient presenting with fever or respiratory or gastrointestinal symptoms suspected for COVID-19. The diagnosis was confirmed by positive real-time reverse transcriptase (RT-PCR) assay for SARS-CoV-2. After the diagnosis, participants were locally randomly assigned by a simple randomization procedure with a 1:1 allocation ratio (computer-generated), to one of two treatment groups: (a) HDx, performed by the use of an MCO membrane (Theranova VR 400- Baxter, Deerfield, IL, USA), or (b) standard bicarbonate dialysis treatment based on the use of a polymetilmetachrilate (PMMA)-based PLD (BG-U, Estor SpA, Pero, Italy).

For dialysis treatments in COVID-19 patients, the use of a blood flow rate (Qb) of 250–300 mL/min was allowed, while in the control group, Qb was fixed at 300 mL/min. For all patients, the dialysate flow rate (Qd) was set at 600 mL/min.

Patients presenting with a history of allergic reactions to dialysis membranes were excluded from randomization. Clinical management decisions were left to the attending physicians. For each patient, we collected: (i) demographics and clinical data, (ii) laboratory and radiological findings, (iii) data on hospitalization, disease duration, and clinical outcomes, and (iv) serum levels of inflammatory markers, such as C-reactive protein (CRP), procalcitonin (PCT), ferritin, lymphocyte count and neutrophil/lymphocyte ratio (N/L). Moreover, we analyzed longitudinal changes in the circulating levels of interleukin-6 (IL-6), interleukin-8 (IL-8), interleukin-10 (IL-10), soluble toll-like receptor 4 (sTLR4), and interferon-gamma (IFN-γ). Maintenance HD patients who tested negative for SARS-CoV2 PCR constituted the control group. The study was performed according to the Declaration of Helsinki and was approved by the local Ethics Committee (N. Registro CER Liguria: 135/2020) and registered on ClinicalTrials.org (NCT04685447). All participants provided written informed consent before the enrollment.

### 2.2. Cytokine Determinations

Blood samples, collected in polypropylene tubes, were centrifuged at 3200 rpm for 10 min. Plasma was separated by centrifugation and stored at −20 °C until it was assayed. Plasma human IL-6, IL-8/CXCL8, IL-10, and IFN-γ levels were determined using the Quantikine ELISAs (R&D Systems, Minneapolis, MN, USA), whereas, for sTLR-4, ELISA, commercially kits were obtained by MyBioSource (D.B.A., Segrate, Italy). The tests were performed according to the manufacturer’s protocol and the results were expressed for all cytokines as pg/mL and sTLR4 as ng/mL. The samples were all run in duplicate. The minimum detectable dose was 0.7 pg/mL for IL-6, 3.5 pg/mL for IL-8/CXCL8, 3.9 pg/mL for IL-10, 8.0 pg/mL for IFN-γ and 0.022 ng/mL for sTLR4. Samples were collected before and after the dialysis treatment in the first session after the diagnosis of COVID-19 (T0), and at one and two weeks after the diagnosis (T7 and T14, respectively). Post-dialysis cytokine levels were corrected for dialysis-induced changes in blood volume by multiplying post-dialysis concentration with the ratio between serum albumin before and after dialysis [17]. The effects of dialysis treatments on cytokine levels were evaluated by the comparison of the reduction rate (RR, i.e., (1 − (corrected PostHD/PreHD)).

### 2.3. Statistical Analysis

The sample size estimation was based on the results of previous studies describing the effects of the two different dialyzers on IL-6, and the highest estimate was considered. IL-6 was reported to be 5.0 ± 1.9 and 9.5 ± 2.15 pg/mL in basal conditions, with a variation from basal of 2 and 3 pg/mL, i.e., a 38% and 30% reduction, after PDL and HDx treatment, respectively [13,14]. Therefore, with a power of 80% and a significance level of 0.05, we estimated that a sample of n = 11 patients per group (22 patients in total) would be needed. Moreover, considering a dropout rate of about 30%, we planned to enroll at least 14 patients in each group. Data are presented as mean ± standard deviation (SD) or interquartile ranges (IQR), if not normally distributed (as evaluated by Shapiro Test). Mixed models for repeated measures, followed by Tukey’s multiple comparison test, Student t-test, or nonparametric Mann–Whitney test, were used to assess the differences among patients affected by COVID-19 and the control group, and among patients undergoing different HD treatments during the study. Proportions for categorical variables were compared using the χ2 test. Spearman-Rho was used to assess the correlations between the different clinical and laboratory variables. Data analysis was performed with GraphPad Prism statistical package (version 8.00, GraphPad Software, San Diego, CA, USA). A two-tailed *p* value < 0.05 was considered statistically significant.

## 3. Results

### 3.1. Clinical Presentation, Radiological Findings and Outcome of COVID-19 in HD Patients

We enrolled 31 HD patients with COVID-19 (68.9 ±15.6 years, 17 males) with a dialysis vintage of 46 (56) months. Twenty-five patients (80%) were hypertensive, 9 (29%) diabetic, 6 (19%) active smokers, and 22 (71%) had a history of cardiovascular disease. The most common initial symptoms were fever (75%), cough (42%), and shortness of breath (27%). For 14 patients, a basal chest X-ray was available, showing: interstitial pneumonia in 7 patients (50%) and lung consolidation in 6 patients (43%). Thirteen patients (42%) were admitted to the hospital because of worsening clinical conditions, while the remaining patients underwent outpatient hemodialysis. In four patients (13%), a superimposed bacterial infection was recognized. Among the hospitalized patients, seven (54%) were treated by non-invasive ventilation, but none were admitted to ICU or underwent tracheal-bronchial intubation. No antiviral treatment was prescribed, while 17 patients (55%) received hydroxychloroquine treatment after the diagnosis of COVID-19. Overall, seven patients (22.5%) died after 15 ± 12 days from COVID-19 diagnosis, whereas 24 patients were shown to be negative from two consecutive RT-PCR assays for SARS-CoV2 after 34 ± 24 days from diagnosis, of whom 21 were alive at the last control of the 31 December 2020, after 283 ± 9 days of follow-up.

### 3.2. Basal Laboratory Characteristics

Biochemical parameters and cytokine levels of HD patients affected by COVID-19 were compared with the control group, which constituted 14 COVID-19 negative HD patients with a mean age of 66.3 ± 15.2 and a dialysis vintage of 28 (50) months (not significant vs. COVID-19 group). None of the patients included in the control group developed COVID-19 within one week after the enrollment in the study

Looking at the actual Qb used in COVID-19 positive patients, we observed that in 26/31 patients (84%) a Qb of 300 mL/min was prescribed, while in the remaining patients a Qb of 250 mL/min was set. However, there were no significant differences in median Qb between COVID-19 patients and the control group.

Compared with the control group, patients affected by COVID-19 presented with a significantly reduced number of white blood cells and lymphocytes and higher levels of ferritin, lactate dehydrogenase (LDH), and PCT. Moreover, COVID-19 patients had a lower CD4, CD8, and natural killer (NK) cell number (*p* < 0.01). The analysis of basal cytokine profile revealed that COVID-19 patients had higher levels of IL-10 (15.2 (12.5) vs. 1.2 (1.4) pg/mL, *p* = 0.02), while the levels of IL-6, IL-8 and sTLR4 were comparable. In COVID-19 patients, IL-6 concentration directly correlated with IL-10 levels (r = 0.48, *p* < 0.01) and CRP levels directly correlated with N/L ratio (r = 0.37, *p* = 0.04). IFN-γ levels were detectable only in five patients (16%) of the COVID-19 group and 3 (21%) of the control group (*p* = 0.7). The main clinical and laboratory characteristics of the evaluated patients are shown in Table 1.

### 3.3. Effects of Dialysis Treatment on Cytokine Removal

After confirmation of the COVID-19 diagnosis, the patients were randomized to the experimental treatments, but two of them were excluded from randomization because of a history of allergic reactions to multiple dialysis membranes. Then, the remaining 29 patients were assigned to HDx (n = 15) or PLD (n =14). Basal clinical characteristics and dialysis parameters, including Qb, laboratory findings and cytokine levels, were not significantly different between the two groups (see Table S1). When we considered the effects of the first treatment with the two experimental dialysis modalities, we found no significant differences between predialysis and post-dialysis IL-6 and sTLR4 concentrations, even when corrected for dialysis-related blood volume changes. Instead, IL-8 levels significantly decreased after HD treatment in both groups. The IL-8 reduction rate was correlated with IL-8 predialysis levels (r = 0.43, *p* = 0.01). Finally, IL-10 levels significantly decreased after HDx (Table 2).

### 3.4. Longitudinal Cytokine Profile According to Dialysis Modalities

**Figure 1:**
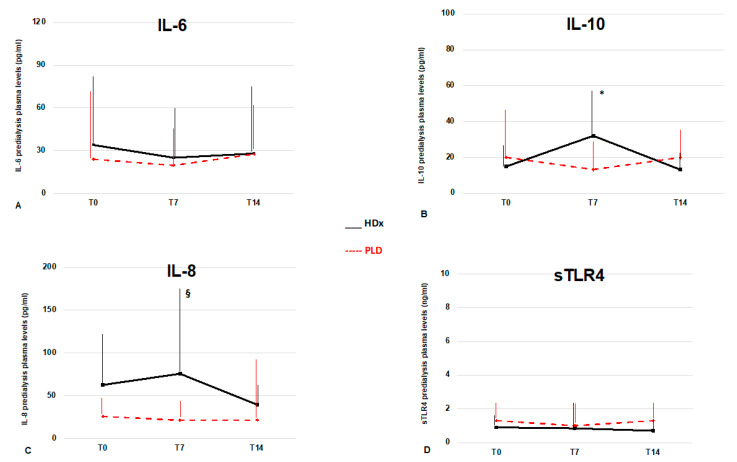
Time trends of pro- and anti-inflammatory cytokines in maintenance HD patients with COVID-19. (**A**) IL-6 did not show significant longitudinal changes during the observation interval. (**B**) IL-10 showed a significant increase in the first week after the diagnosis of COVID-19 in the HDx group. (**C**) IL-8 levels did not show significant intra-group changes, but were higher in patients undergoing HDx at T7. (**D**) sTLR4 levels did not show significant modifications during the study. Black lines for expanded hemodialysis (HDx) and red lines for protein-leaking dialysis (PLD). * = *p* < 0.05 vs. T0; § = *p* < 0.05 vs. PLD.

In patients randomized to experimental treatments circulating cytokine levels were evaluated at one and two weeks from diagnosis. In the HDx group, one patient died, and three patients were transferred to other centers during the first week; subsequently, a second death occurred during the second week. Therefore, at T14 control, we analyzed the data from 10 patients. At T7 control, inflammatory markers were unchanged, while among the cytokines, only IL-10 showed a significant increase with respect to T0 (*p* < 0.05). At T14 control, we found CRP and PCT levels significantly decreased compared with previous values (*p* < 0.05), whereas cytokine levels were unchanged, apart from a non-significant decrease of IL-8 and IL-10 levels. In the PLD group, three patients died during the first week. We did not observe any further drop-outs until the end of the study, and therefore we analyzed the data from 11 patients at T14 control (Table 3). During the observation interval, there was a significant increase in CD4 cell count (*p* < 0.05), while at T14 control, the N/L ratio decreased when compared to T0 (*p* = 0.03). No significant changes in cytokine levels occurred. Overall, we observed no significant differences between the two groups of treatments in the laboratory and inflammatory parameters at different time points, except for IL-8 levels, which were higher in the HDx group than in PLD at T7 control (76 (99) vs. 21.5 (22) pg/mL, *p* = 0.007) (Figure 1). Finally, IFN-γ was detectable in only four patients of the PLD group and one patient of the HDx group at T0 and undetectable at the following controls.

### 3.5. Cytokine Levels and Clinical Outcomes

Looking at clinical outcomes, in terms of hospitalization and death, we did not find significant differences in cytokine levels (see Table S2). Similarly, there were no significant differences in clinical outcomes between the patients undergoing HDx or PLD (see Table S3).

## 4. Discussion

In this study, we attempt to give a response to two crucial questions, i.e., if inflammation occurs in maintenance HD patients affected by COVID-19 and if dialysis treatment may be useful to modulate immunity and inflammatory responses in this setting. These issues were raised from ongoing evidence of a strict correlation between immune response, inflammation, and the clinical course of COVID-19 [18,19]. To provide a complete profile of inflammatory response during COVID-19, we evaluated prospective changes of general inflammatory markers, such as CRP, PCT, and ferritin, together with the different pro-and anti-inflammatory cytokines implicated in both innate and adaptive immunity. First, we studied basal levels and longitudinal changes of IL-6, which is produced by many types of cells and has well-characterized pro-inflammatory properties [20]. This molecule has been advocated as one of the main components of the cytokine storm found in COVID-19 patients, and its levels have correlated with disease severity and unfavorable outcomes [21]. We also evaluated the circulating levels of IL-8, which may amplify inflammatory processes, mainly acting as a chemoattractant for neutrophils, and promoting phagocytosis [22]. In contrast, IL-10 has classically been designated an immunosuppressive cytokine, firstly characterized as a T helper 2 specific cytokine, then also associated with T regulatory cell responses [23]. Moreover, since toll-like receptors (TLRs) have a primary role in promoting an innate immune response against pathogens, including SARS-CoV2 [24], we studied TLR pathways by the analysis of the sTLR4, accounting for the soluble form of extracellular TLR4 domain. sTLR4 acts as a potent inhibitor of TLR4-mediated inflammation, and in humans, its serum levels are elevated in different conditions, like sepsis and some inflammatory diseases. In the specific setting of HD patients, circulating sTLR4 levels have been found to correlate with inflammatory and nutritional parameters [25]. Finally, we evaluated the level of IFN-γ, which plays an essential role in influencing the growth and differentiation of immunocompetent lymphocytes [26]. Overall, looking at these different molecules, together with other inflammatory markers, we found that HD patients affected by COVID-19, when compared with HD patients without COVID-19, showed some features commonly reported in general COVID-19 patients, including lymphopenia and high levels of ferritin and LDH, associated with an increase in the levels of anti-inflammatory IL-10. Then, we observed no remarkable changes in cytokine levels during the disease course (with almost undetectable IFN-γ levels), independently from the clinical outcome. An exception may by IL-8 and IL-10, which showed a trend to increase within the first week after COVID-19 diagnosis in the HDx group. However, considering that the absolute levels of many cytokines were not so dissimilar between COVID-19 patients and controls, and mainly evaluating the time trend of inflammatory molecules, it appears that HD patients had a blunted immune response to COVID-19. The lack of a marked elevation of inflammatory cytokines during COVID-19 in HD patients is not surprising and may represent an expression of the so-called inflammaging [27]. This phenomenon refers to the premature aging of the immune system found in HD patients, and it is characterized by the chronic activation of the immune system, increased oxidative stress, and low-grade systemic inflammation [28]. This condition, whose pathogenesis has been related to the uremic environment and bioincompatibility of dialysis membranes, makes HD patients less prone to adequately respond to infectious injury [29,30]. Interestingly, in the setting of COVID-19, the blunted immune response found in HD patients could have a double significance. Indeed, while it may account for the high susceptibility of this population to the infection, on the other hand, considering that the hyperinflammatory syndrome is related to the disease severity, it could justify the clinical observation that most HD patients with COVID-19 have mild symptoms [31]. Aiming to define whether dialysis treatment may be considered a therapeutic tool to modulate the inflammatory response in COVID-19, we also investigated the effects of two dialysis modalities on cytokine levels. Then, we evaluated dialysis membranes that have shown the ability to remove inflammatory molecules by two different mechanisms. Therefore, we tested HDx performed by an MCO dialyzer that, due to the presence of tight pore size distribution on the membrane, guarantees significant convection, balanced by an internal back-filtration, allowing an improved removal of middle-to-high weight range molecules, with marginal or no albumin leak [15,32]. This approach was compared with dialysis performed using a PLD dialyzer (namely, PMMA), which combining high-flux membrane characteristics with a high absorption capacity, can reduce the circulating levels of toxins and cytokines [33]. Then, we considered the effect of a single treatment on inflammatory cytokines, evaluating the pre-dialysis and post-dialysis concentration of each molecule. We observed that HD treatment mainly affected IL-8 levels, which were significantly reduced after the dialysis in both groups of patients. Interestingly, in agreement with previous studies, we found that intradialytic IL-8 reduction was correlated with pre-dialysis IL-8 levels, thus confirming that the concentration gradient is the dominant factor influencing IL-8 removal during hemodialysis [34]. Moreover, in the HDx group, IL-10 also showed significant dialysis removal. Looking at longitudinal changes of cytokine levels between the two treatment groups, we found that patients on HDx showed an increase in IL-8 and IL-10 levels one week after the COVID-19 diagnosis. Therefore, it seems that the cytokine removal performed by HDx was associated with a mild inflammatory activation accompanied by a partial compensatory reaction. A causal relationship of this finding remains to be proven. However, with the limits due to the small sample size of our cohort, no other significant changes in cytokine levels occurred at the following controls, whereas some mild changes were found in other inflammatory markers, such as CRP and N/L ratio, thus confirming that overt inflammation is not present in maintenance HD patients with COVID-19. We are aware of the methodological limitations of this study, including that the enrollment of control subjects after only one negative SARS-CoV-2 PCR test made possible the inclusion of false-negative cases (even if none of the patients of our control cohort developed COVID-19 immediately after the enrollment).

Moreover, we evaluated dialysis-dependent cytokine changes evaluating RRs that can be influenced by the distribution volume of each cytokine. Indeed, we could not perform a whole evaluation of cytokine kinetic parameters, which could have been more informative.

Similarly, an extended evaluation of the trend of the inflammatory cytokines, and its association with other factors, such as the generation of antibody response, could provide additional useful data.

Finally, the limited sample size of the studied population may not have shown small differences between pre-and post-dialysis cytokine levels, such as between the two groups of treatment. So, the negative results of this study should be interpreted carefully and need confirmation in larger investigations. Finally, the small sample size did not allow us to perform sub-analysis (for example, evaluating the clinical outcomes according to the cytokine circulating levels).

Nevertheless, we think that our data focus on a crucial aspect of the pathogenesis of COVID-19 in the HD population, which may have therapeutic implications. In particular, we suggest that in maintenance HD patients, SARS-CoV2 infection is not related to a sustained inflammatory response during the disease course. Hence, the modulation of the inflammation seems not to be a suitable therapeutic target in this specific patient population. Coherently, the use of dialysis membranes, which have previously shown effectiveness in removing pro-inflammatory molecules, has a minimal impact on cytokine profile and no correlation with the clinical outcome. These findings imply that, in the absence of an acute inflammatory response, other pathophysiological mechanisms should be investigated to explain the high mortality of COVID-19 observed in maintenance HD patients [35]. Finally, anti-inflammatory and immunosuppressive drugs (such as, corticosteroids or IL-6 inhibitors) should be used with caution in HD patients who present with an impaired immune system.

## Figures and Tables

**Table 1:** Clinical and laboratory parameters of COVID-19 positive HD patients at diagnosis (T0) compared with COVID-19 negative HD patients.

	COVID-19 HD	NO COVID-19 HD	*p*
N	31	14	
Age, years	68.9 ± 15.6	66.3 ± 15.2	0.5
Sex, M/F	17/14	9/5	0.5
Dialysis vintage, months	46 (56)	28 (50)	0.86
Qb, mL/min	292 ± 18	300	0.12
Diabetes, N (%)	7 (27)	5 (35)	0.71
Hypertension, N (%)	21 (77)	10 (70)	0.7
CVD disease, N (%)	20 (72)	11 (78)	0.9
WBC, ×10^9^/L	5.8 ± 3.6	7.9 ± 2.3	0.003
Neutrophils, ×10^9^/L	4.5 ± 3.6	5.5 ± 2.3	0.04
Lymphocytes, ×10^9^/L	0.7 ± 0.36	1.4 ± 0.6	<0.0001
N/L ratio	5.5 (5)	3.5 (3.6)	0.2
CRP, mg/L	27.5 (45)	7.25 (19.8)	0.29
Ferritin, µg/L	500 (904)	158 (165)	0.001
LDH, U/L	238 ± 69	182.2 ± 40	0.01
Fibrinogen, g/L	4.8 (4.9)	4.2 (3.1)	0.2
PCT, µg/L	1.6 (4.3)	0.6 (0.48)	0.02
CD3+CD4+, cells/µL	283 ± 158	781 ± 378	0.001
CD3+CD8+, cells/µL	150 (133)	356 (390)	0.002
CD19+, cells/µL	59 (68)	189 (189)	0.15
NK, cells/µL	83(132)	163 (131)	0.02
IL-6, pg/mL	24 (43)	12 (53)	0.1
IL-8, pg/mL	35.1 (100)	24.1 (234)	0.9
IL-10, pg/mL	15.2 (12.5)	1.2 (1.4)	<0.001
sTLR4, ng/mL	1 (1.1)	1.1 (1.6)	0.16

Data are expressed as mean ± SD or median (IQR). All values were determined in predialysis. Abbreviations: White blood cells (WBC), neutrophil/lymphocyte (N/L), C-reactive protein (CRP), lactate dehydrogenase (LDH), procalcitonin (PCT), neutrophil/lymphocyte ratio (N/L)., interleukin-6 (IL-6), interleukin-8 (IL-8), interleukin-10 (IL-10), soluble TLR4 (sTLR4).

**Table 2:** Effects of a single treatment with different dialysis membranes on cytokine removal in maintenance HD patients affected by COVID-19.

	HDx (n,15)				PLD (n,14)			
	Pre-HD	Post-HD	*p*	RR	Pre-HD	Post-HD	*p*	RR
IL-6, pg/mL	34 (67)	39.5 (39)	0.5	0.1 (0.83)	24 (51.5)	27.4 (51.6)	0.58	−0.02 (0.49)
IL-8, pg/mL	62.8 (59.5)	18.5 (35.8)	<0.001	0.5 (0.46)	26 (20)	18.7 (10.3)	0.001	0.28 (0.49)
IL-10, pg/mL	14.9 (12)	10.6 (8.6)	0.03	0.29 (0.54)	20 (17.6)	9 (5.1)	0.1	0.27 (0.4)
sTLR4, ng/mL,	0.9 (0.7)	0.92 (1.4)	0.6	0.06 (0.5)	1.3 (1.1)	0.88 (0.8)	0.2	0.15 (0.9)
Albumin, g/dL	3.3 ± 0.5	3.6 ± 0.6	<0.01	−0.06 (0.036)	3.4 ± 0.3	3.8 ± 0.4	<0.01	−0.1 (0.05)

Data are expressed as mean±SD or median (IQR). Abbreviations: Pre dialysis (Pre-HD), post dialysis (Post-HD), expanded hemodialysis (HDx), protein-leaking dialysis (PLD), hemodialysis (HD), reduction rate (RR), interleukin-6 (IL-6), interleukin-8 (IL-8), interleukin-10 (IL-10), soluble TLR4 (sTLR4). Post-HD levels of cytokines were corrected for dialysis-induced changes in blood volume by multiplying post dialysis concentration with the ratio between serum albumin before and after dialysis [17].

**Table 3:** Longitudinal changes of biochemical parameters and inflammatory cytokines in COVID-19 patients treated with two different dialysis membranes.

HDx					PLD			
	T0	T7	T14	*p*	T0	T7	T14	*p*
N	15	11	10		14	11	11	
WBC, × 10^9^/L	5.9 ± 3.3	5.8 ± 2.7	6.4 ± 2.6	0.9	6 ± 4.3	5.7 ± 1.5	5.5 ± 2.3	0.34
Neutrophils, × 10^9^/L	4.7 ± 3.1	4.5 ± 2.6	4.1 ± 3	0.13	4.4 ± 4.3	4.2 ± 1.3	2.5 ± 1.5	0.09
Lymphocytes, × 10^9^/L	0.6 ± 0.24	0.73 ± 0.26	0.63 ± 0.41	0.6	0.86 ± 0.4	0.85 ± 0.4	0.8 ± 0.4	0.7
N/L ratio	7.1 (7.1)	5.2 (7.8)	6.4 (7.9)	0.7	4.6 (4.5)	5.3 (3.2)	3 (2.2) *	* 0.03 vs. T0
CRP, mg/L	15.3 (41.7)	13.1 (19)	11.4 (15.9) *	* 0.02 vs. T0,T7	24 (64)	12.2 (15)	24.5 (45)	0.33
Ferritin, µg/L	638 (903)	717 (629)	433 (586)	0.6	308 (1008)	358 (1036)	554 (2485)	0.48
LDH, U/L	230 ± 75	193 ± 34	206 ± 41.8	0.2	253 ± 65	247 ± 70	283 ± 135	0.54
PCT, µg/L	1.8 (8.3)	0.7 (2.7)	0.72 (0.7) *	* 0.009 vs. T0	2 (3.7)	1 (1.4)	1.1 (1.45)	0.5
CD3+CD4+cells/µL	282 ± 173	430 ± 133	410 ± 182	0.08	283 ± 147	478 ± 291	432 ± 222	0.04
CD3+CD8+cells/µL	160 ± 128	202 ± 81,8	202 ± 81,8	0.7	227 ± 113	326 ± 189	280 ± 117	0.24
CD19+, cells/µL	63.7 ± 37	47.5 ± 48.4	60.6 ± 41	0.38	51.4 ± 40	54.3 ± 39.7	56 ± 50	0.8
NK, cells/µL	87.5 (103.5)	78 (218)	117 (73)	0.7	82 (645)	127 192)	148 (235)	0.26
IL-6, pg/mL	34 (67)	25 (47.5)	28.1 (68.9)	0.6	24 (51.5)	19.6 (29.8)	27.6 (32)	0.4
IL-8, pg/mL	62.8 (59.5)	76 (99) §	39.6 (22.7)	0.27	26 (20)	21.5 (22)	21.8 (69)	0.6
IL-10, pg/mL	14.9 (12.4)	32 (26.9) *	13.1 (12.6)	* 0.04 vs. T0	20 (24)	13.2 (17.8)	19.9 (18.2)	0.65
sTLR4, ng/mL	0.9 (0.7)	0.86 (1.7)	0.7 (0.75)	0.48	1.3 (1.1)	1 (1.3)	1.3 (1.1)	0.26
IFN-γ, pg/mL	0.1 (0.1)	0.1 (0)	0.1 (0)	-	0.1 (0.05)	0.1 (0)	0.1 (0)	-

Data are expressed as mean±SD or median (IQR). All values were determined in predialysis. Comparisons were performed by mixed models for repeated measures, followed by Tukey’s multiple comparison test. Abbreviations: Expanded hemodialysis (HDx), protein-leaking dialysis (PLD), white blood cells (WBC), neutrophil/lymphocyte ratio (N/L), C-reactive protein (CRP), lactate dehydrogenase (LDH), procalcitonin (PCT), neutrophil/lymphocyte ratio (N/L)., interleukin-6 (IL-6), interleukin-8 (IL-8), interleukin-10 (IL-10), soluble TLR4 (sTLR4), and interferon-gamma (IFN-γ). § = *p* < 0.05 vs. IL-8 T7 PLD. * *p* values are reported in the related columns.

## Data Availability

The data underlying this article are available in Harvard Dataverse, at https://doi.org/10.7910/DVN/LI7WZ2 (accessed on 26 March 2021).

## Supplementary Materials

The following are available online at https://www.mdpi.com/article/10.3390/jcm10071383/s1, Table S1: Basal characteristics of study cohort at the beginning of treatment with two different dialysis membranes., Table S2: Basal cytokine levels and clinical outcomes in HD patients with COVID-19; Table S3: Clinical outcomes of HD patients with COVID-19 treated with two different dialysis membranes.
