# Supplementary material for: Effects of Different Dialysis Strategies on Inflammatory Cytokine Profile in Maintenance Hemodialysis Patients with COVID-19: A Randomized Trial
Source: J Clin Med. 2021 Mar 30;10(7):1383. doi: 10.3390/jcm10071383 (PMC8037035; doi:10.3390/jcm10071383)
Supplement: Supplementary file 1 [file jcm-10-01383-s001.pdf]

EFFECTS OF DIFFERENT DIALYSIS STRATEGIES ON INFLAMMATORY CYTOKINE PROFILE IN MAINTENANCE HEMODIALYSIS PATIENTS WITH COVID-19: A RANDOMIZED TRIAL.

Supplementary table 1.
Basal characteristics of study cohort at the beginning of treatment with two different dialysis membranes

	HDx	PLD	p
**N**	15	14	
**Age, years**	71.2±15.4	64.2±15.4	0.54
**Sex, M/F**	8/7	7/7	0.9
**Dialysis vintage, months**	38 (73)	54 (51)	0.7
**Qb, mL/min**	290±20	293±18	0.7
**WBC, x10^9/L**	5.9±3.3	6±4.3	0.88
**Neutrophils, x10^9/L**	4.7±3.2	4.4±4.3	0.85
**Lymphocytes, x10^9/L**	0.6±0.24	0.86±0.4	0.18
**N/L ratio**	7.1 (7.8)	4.6 (4.5)	0.14
**CRP, mg/L**	15.3 (41.7)	24 (64)	0.4
**Ferritin, μg/L**	638 (903)	308 (1008)	0.24
**LDH, U/L**	230±75	253±65	0.19
**PCT, μg/L**	1.8 (8.3)	2 (3.7)	0.85
**CD3+CD4+, cells/μL**	284±173	283±147	0.99
**CD3+CD8+ , cells/μL**	162±128	227±113	0.17
**CD19+, cells/μL**	60.6±37	51.4±40	0.54
**NK, cells/μL**	87.5 (103.5)	82 (645)	0.88
**IL-6, pg/ml**	34 (67)	24 (51.5)	0.4
**IL-8, pg/ml**	62.8 (59.5)	26 (20)	0.22
**IL-10, pg/ml**	14.9 (12)	20 (24)	0.35
**sTLR4, ng/ml**	0.9 (0.7)	1.3 (1.1)	0.16
**IFN-γ, pg/ml**	0.1 (0.1)	0.1 (0.05)	0.5

Data are expressed as mean ± SD or median (IQR). All values were determined in predialysis.
Abbreviations: Expanded hemodialysis (HDx), protein-leaking dialysis (PLD), white blood cells (WBC), neutrophil/lymphocyte (N/L), C-reactive protein (CRP), lactate dehydrogenase (LDH), procalcitonin (PCT), neutrophil/lymphocyte ratio (N/L)., interleukin-6 (IL-6), interleukin-8 (IL-8), interleukin-10 (IL-10), soluble TLR4 (sTLR4), and interferon-gamma *(IFN-γ)*

Supplementary table 2.
Basal cytokine levels and clinical outcomes in HD patients with COVID-19

	**Hospitalized patients**			**Survival status**		
	**Yes (13)**	**No (18)**	**p**	**Died (7)**	**Alive(24)**	**p**
**IL-6, pg/ml**	48 (74)	23.1(31)	0.2	45 (45.7)	20.7 (48.5)	0.1
**IL-8, pg/ml**	26 (43.8)	40 (210)	0.3	33 (43)	26 (100)	0.91
**IL-10, pg/ml**	14.9 (21)	6.1 (18)	0.08	9 (29)	10 (19)	0.9
**sTLR4, ng/ml**	0.98 (0.9)	1.1 (1.2)	0.3	1(1)	1 (1.2)	0.9

Data are expressed as median (IQR). Abbreviations: interleukin-6 (IL-6), interleukin-8 (IL-8), interleukin-10 (IL-10), soluble TLR4 (sTLR4),

Supplementary table 3.
Clinical outcomes of HD patients with COVID-19 treated with two different dialysis membranes

	**HDx**	**PLD**	**p**
**N**	15	14	
**Illness duration, days ***	34±28	30±22	0.9
**Hospitalized patients, n (%)**	8 (53%)	5 (36%)	0.46
**Deaths, n (%)**	2 (13%)§	3 (21%)	0.65

Abbreviations: Expanded hemodialysis (HDx), protein-leaking dialysis (PLD)
* Illness duration was calculated from the date of the first positive RT-PCR assay for SARS-COV2 infection to the date of death or of the two consecutive negative RT-PCR assays.
§ In HDx group two additional deaths occurred after the completion of the study of cytokine profile, respectively at 28 and 38 days from COVID-19 diagnosis.
